# Temporal trends and sociodemographic determinants of time to nephrectomy in localized renal cell cancer

**DOI:** 10.1007/s11255-026-05303-0

**Published:** 2026-07-23

**Authors:** Maximilian Filzmayer, Michele Petix, Leonardo Quarta, Filippo Orlandi, Jordan A. Goyal, Luca Carmignani, Alberto Briganti, Salvatore Micali, Shahrokh F. Shariat, Mike Wenzel, Miriam I. Traumann, Clara Humke, Fred Saad, Felix K.-H. Chun, Pierre I. Karakiewicz

**Affiliations:** 1 Cancer Prognostics and Health Outcomes Unit, Division of Urology, University of Montreal Health Center, Montreal, Canada; 2 Department of Urology, Goethe University Frankfurt, University Hospital, Frankfurt am Main, Germany; 3 Università degli Studi di Milano, Milan, Italy; 4 Department of Urology, IRCCS Policlinico San Donato, Milan, Italy; 5 Vita-Salute San Raffaele University, Milan, Italy; 6 Division of Experimental Oncology/Unit of Urology, Urological Research Institute, IRCCS San Raffaele Scientific Institute, Milan, Italy; 7 Department of Urology, AOU di Modena, University of Modena and Reggio Emilia, Modena, Italy; 8 Department of Urology, Comprehensive Cancer Center, Medical University of Vienna, Vienna, Austria; 9 Department of Urology, Weill Cornell Medical College, New York, USA; 10 Department of Urology, University of Texas Southwestern Medical Center, Dallas, USA; 11 Hourani Center of Applied Scientific Research, Al-Ahliyya Amman University, Amman, Jordan

**Keywords:** Kidney cancer, Nephrectomy, Time to surgical treatment, Trends, Determinants

## Abstract

**Introduction:**

Prolonged time to nephrectomy (TTN) has been associated with adverse oncologic outcomes. We evaluated temporal trends and sociodemographic determinants of prolonged TTN in surgically treated patients with localized renal cell carcinoma (RCC).

**Methods:**

Patients with T1–2 N0 M0 RCC treated with partial (PN) or radical nephrectomy (RN) were identified within the Surveillance, Epidemiology and End Results (SEER) database (2004–2022) and stratified according to TTN ≤ 3 vs. > 3 months. Multivariable logistic regression models identified independent and combined sociodemographic determinants of TTN > 3 months.

**Results:**

Among 54,365 patients, TTN > 3 months was recorded in 28% of PN and 18% of RN patients. The proportion of TTN > 3 months increased over time in both PN (from 19 to 36%, p < 0.001) and RN (from 11 to 31%, p < 0.001). Higher rates of TTN > 3 months were recorded in non-Caucasian vs. Caucasian patients (PN: 33% vs 25%, OR 1.49; RN: 24% vs 15%, OR 1.74), unmarried vs. married individuals (PN: 33% vs. 25%, OR 1.43; RN: 22% vs. 15%, OR 1.58), and males vs. females (PN: 28% vs 27%, OR 1.18; RN: 18% vs 18%, OR 1.20; all p < 0.001). Combined assessment revealed highest rates in non-Caucasian unmarried males at PN (40%, OR 2.70, p < 0.001) and RN (28%, OR 3.09, p < 0.001) compared to lowest rates in Caucasian married females (PN: 21%; RN: 12%).

**Conclusions:**

TTN > 3 months proportion increased over time in localized RCC. In PN and RN, non-Caucasian unmarried males are at highest risk of TTN > 3 months. Therefore, specific focus on these highest risk individuals may be warranted within strategies aimed at minimizing TTN.

**Supplementary Information:**

The online version contains supplementary material available at https://doi.org/10.1007/s11255-026-05303-0.

## Introduction

Time to surgical treatment, defined as the time interval between initial diagnosis and surgery, is important to consider in patients awaiting oncologic care, since longer delays have been associated with adverse prognostic outcomes [1–9]. Prior studies have demonstrated increasing surgical wait times across multiple primaries and have identified sociodemographic predictors of prolonged time to surgical treatment [1, 2].

These considerations also apply to renal cell carcinoma (RCC) patients awaiting nephrectomy, where prolonged time to nephrectomy (TTN) is associated with worse cancer control outcomes [10–18]. Despite this evidence, contemporary population-based analyses evaluating temporal trends in TTN and the impact of sociodemographic characteristics on prolonged TTN among surgically treated patients have not been completed.

We addressed these knowledge gaps using a large population-based cohort of patients with localized (T1–2 N0 M0) RCC from the Surveillance, Epidemiology, and End Results (SEER) database (2004–2022) [19]. We hypothesized that TTN has increased over time and that sociodemographic characteristics represent independent determinants of prolonged TTN. Moreover, we postulated that their combined assessment may provide improved stratification, enabling identification of individuals at particularly high risk for prolonged TTN.

## Materials and methods

### Data source, study population and outcomes of interest

Within the SEER database (2004–2022), we identified adult patients with histologically confirmed localized (T1–2 N0 M0) RCC (International Classification of Diseases for Oncology Third Edition [ICD-O-3] site code C64.9) treated with either partial (PN) or radical nephrectomy (RN) [19–22]. TTN was derived from the SEER variable “Time from diagnosis to treatment (days)”, which records the interval between diagnosis and first treatment with day-level resolution [19]. Patients with missing information on TTN or key patient and tumor characteristics were excluded.

The primary outcome of interest was TTN > 3 vs. ≤ 3 months. This threshold has been applied in several prior RCC analyses, in which TTN > 3 months was associated with increased cancer-specific mortality [12–17]. We evaluated temporal trends in the proportion of patients with TTN > 3 months and assessed sociodemographic determinants of TTN > 3 months, including race/ethnicity (Caucasian vs. non-Caucasian), marital status (married vs. unmarried), and sex (female vs. male).

### Statistical analyses

Separate analyses following identical analytical steps were applied to patients treated with PN or RN. First, temporal trends in the annual proportions of patients with TTN > 3 months were assessed using estimated annual percentage change (EAPC) derived from log-linear regression models. Second, multivariable logistic regression models were fitted to identify sociodemographic determinants of TTN > 3 vs. ≤ 3 months. Sociodemographic variables, namely race/ethnicity, marital status, and sex, were first examined as independent predictors. Subsequently, a composite variable combining these characteristics was generated, resulting in eight distinct groups, to assess their joint effect and potential for improved risk stratification. Covariates included year of diagnosis (continuous), age (continuous), income (low [annual income below the median of 80,000 USD] vs. high), area of residence (urban vs. rural), and tumor size (continuous). Third, sensitivity analyses were performed using alternative TTN thresholds (> 2 and > 4 months) to assess the robustness of the primary definition of prolonged TTN and to enhance the generalizability of the findings. Additionally, an upper truncation at 6 months (TTN > 3–6 vs. ≤ 3 months) was applied to limit the influence of extreme TTN values.

R software was used for statistical computing and graphics (R version 4.4.1, The R Foundation for Statistical Computing, Vienna, Austria). All tests were two-sided with significance set at p < 0.05 following Bonferroni adjustment.

## Results

### Temporal trends of TTN > 3 months in PN or RN

**Fig. 1:**
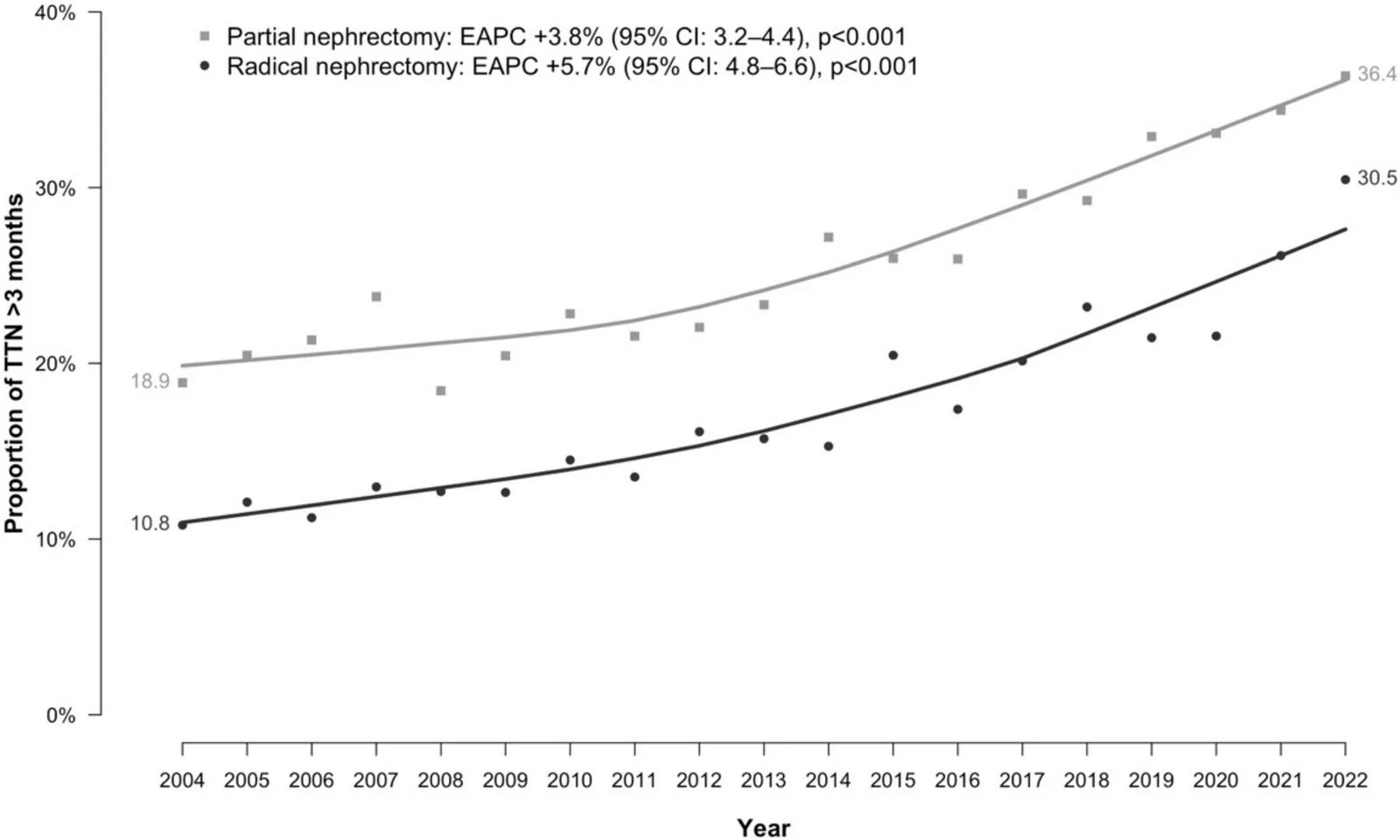
Proportion of time to nephrectomy > 3 months over time among patients with localized renal cell carcinoma (SEER 2004–2022), with estimated annual percentage change (EAPC)

Within the SEER database (2004–2022) we identified 54,365 T1–2 N0 M0 RCC patients treated with nephrectomy (Table 1). Of those, PN accounted for 26,651 (49.0%) patients, and RN for 27,714 (51.0%). TTN > 3 months was recorded in 7428 (27.9%) patients treated with PN, and in 4976 (18.0%) patients treated with RN. During the study span, the proportion of TTN > 3 months significantly increased at both PN (p < 0.001) and RN (p < 0.001; Fig. 1). In PN, the proportion of TTN > 3 months was 18.9% in 2004 vs. 36.4% in 2022 (EAPC + 3.8%). In RN, the proportion of TTN > 3 months was 10.8% in 2004 vs. 30.5% in 2022 (EAPC + 5.7%).

**Table 1:** Descriptive characteristics of patients with localized renal cell carcinoma (SEER 2004–2022)

Characteristics	Overall, n = 54,365	Partial nephrectomy, n = 26,651 (49.0%)	Radical nephrectomy, n = 27,714 (51.0%)
Year of diagnosis, median (IQR)	2016 (2011, 2019)	2016 (2012, 2019)	2015 (2009, 2018)
Age (years), median (IQR)	62 (53, 70)	61 (52, 68)	63 (54, 71)
Caucasian race/ethnicity, n (%)	35,501 (65.3%)	17,481 (65.6%)	18,020 (65.0%)
Married, n (%)	35,095 (64.6%)	17,547 (65.8%)	17,548 (63.3%)
Male sex, n (%)	34,078 (62.7%)	16,834 (63.2%)	17,244 (62.2%)
Low income (< 80,000 USD per year), n (%)	27,654 (50.9%)	12,381 (46.5%)	15,273 (55.1%)
Urban residence, n (%)	47,215 (86.8%)	23,646 (88.7%)	23,569 (85.0%)
Tumor size (cm), median (IQR)	3.8 (2.6, 5.5)	3.0 (2.2, 4.0)	5.0 (3.5, 7.0)
T substage, n (%)			
T1a	30,535 (56.2%)	20,416 (76.6%)	10,119 (36.5%)
T1b	16,831 (31.0%)	5578 (20.9%)	11,253 (40.6%)
T2a	4994 (9.2%)	540 (2.0%)	4454 (16.1%)
T2b	2005 (3.7%)	117 (0.4%)	1888 (6.8%)
Time to nephrectomy > 3 months, n (%)	12,404 (22.8%)	7428 (27.9%)	4976 (18.0%)

### Sociodemographic determinants of TTN > 3 months in PN

In patients treated with PN, TTN > 3 months was more frequent in non-Caucasian vs. Caucasian patients (33.4% vs. 25.0%, p < 0.001), in unmarried vs. married individuals (32.6% vs. 25.4%, p < 0.001), and in males vs. females (28.4% vs. 26.9%, p = 0.009, Table 2), in that descending order. In multivariable logistic regression models, all three sociodemographic risk variables, namely non-Caucasian race/ethnicity (odds ratio [OR] 1.49, 95% confidence interval [CI] 1.41–1.58, p < 0.001), unmarried status (OR 1.43, 95% CI 1.35–1.52, p < 0.001), and male sex (OR 1.18, 95% CI 1.11–1.25, p < 0.001), achieved independent predictor status for TTN > 3 months (Table 3).

**Table 2:** Stratification according to time to nephrectomy ≤ 3 months vs. > 3 months of patients with localized renal cell carcinoma (SEER 2004–2022)

Characteristics	Partial nephrectomy			Radical nephrectomy		
	TTN ≤ 3 months^a^	TTN > 3 months^a^	p-value^b^	TTN ≤ 3 months^a^	TTN > 3 months^a^	p-value^b^
Overall	19,223 (72.1%)	7428 (27.9%)		22,738 (82.0%)	4976 (18.0%)	
Year of diagnosis	2016 (2012, 2019)	2017 (2013, 2020)	< 0.001	2014 (2009, 2018)	2016 (2012, 2019)	< 0.001
Age (years)	60 (51, 68)	61 (53, 69)	< 0.001	62 (54, 71)	64 (56, 72)	< 0.001
Race/ethnicity			< 0.001			< 0.001
Caucasian	13,113 (75.0%)	4368 (25.0%)		15,326 (85.1%)	2694 (14.9%)	
Non-Caucasian	6110 (66.6%)	3060 (33.4%)		7412 (76.5%)	2282 (23.5%)	
Marital status			< 0.001			< 0.001
Married	13,084 (74.6%)	4463 (25.4%)		14,853 (84.6%)	2695 (15.4%)	
Unmarried	6139 (67.4%)	2965 (32.6%)		7885 (77.6%)	2281 (22.4%)	
Sex			0.009			0.3
Female	7173 (73.1%)	2644 (26.9%)		8623 (82.4%)	1847 (17.6%)	
Male	12,050 (71.6%)	4784 (28.4%)		14,115 (81.9%)	3129 (18.1%)	
Income			0.1			0.1
≥ 80,000 USD per year	10,098 (70.8%)	4172 (29.2%)		10,138 (81.5%)	2303 (18.5%)	
< 80,000 USD per year	9125 (73.7%)	3256 (26.3%)		12,600 (82.5%)	2673 (17.5%)	
Area of residence			0.1			0.8
Rural	2209 (73.5%)	796 (26.5%)		3398 (82.0%)	747 (18.0%)	
Urban	17,014 (72.0%)	6632 (28.0%)		19,340 (82.1%)	4229 (17.9%)	
Tumor size (cm)	3.0 (2.2, 4.0)	3.0 (2.2, 4.0)	< 0.001	5.0 (3.5, 7.0)	4.4 (3.2, 6.0)	< 0.001

^a^median (IQR), n (%)

^b^Wilcoxon rank sum test, Pearson’s chi-squared test

**Table 3:** Multivariable logistic regression models of sociodemographic characteristics associated with time to nephrectomy > 3 months (vs. ≤ 3 months) in patients with localized renal cell carcinoma (SEER 2004–2022), individually and in combination

Time to nephrectomy > 3 months (vs. ≤ 3 months)^a^	Partial nephrectomy			Radical nephrectomy		
	OR	95% CI	p-value	OR	95% CI	p-value
Male sex (Ref. female sex)	1.18	1.11–1.25	< 0.001	1.20	1.12–1.28	< 0.001
Unmarried (Ref. married)	1.43	1.35–1.52	< 0.001	1.58	1.48–1.69	< 0.001
Non-Caucasian race/ethnicity (Ref. Caucasian)	1.49	1.41–1.58	< 0.001	1.74	1.63–1.86	< 0.001
Caucasian married females	Ref			Ref		
Caucasian married males	1.20	1.10–1.33	< 0.001	1.14	1.02–1.29	0.029
Caucasian unmarried females	1.44	1.28–1.62	< 0.001	1.59	1.38–1.83	< 0.001
Caucasian unmarried males	1.71	1.53–1.91	< 0.001	2.06	1.80–2.36	< 0.001
Non-Caucasian married females	1.63	1.44–1.86	< 0.001	1.86	1.59–2.18	< 0.001
Non-Caucasian married males	1.70	1.53–1.89	< 0.001	2.18	1.92–2.49	< 0.001
Non-Caucasian unmarried females	2.07	1.82–2.35	< 0.001	2.64	2.29–3.05	< 0.001
Non-Caucasian unmarried males	2.70	2.38–3.06	< 0.001	3.09	2.67–3.57	< 0.001

^a^adjusted for year of diagnosis, age, income, area of residence, and tumor size

**Fig. 2:**
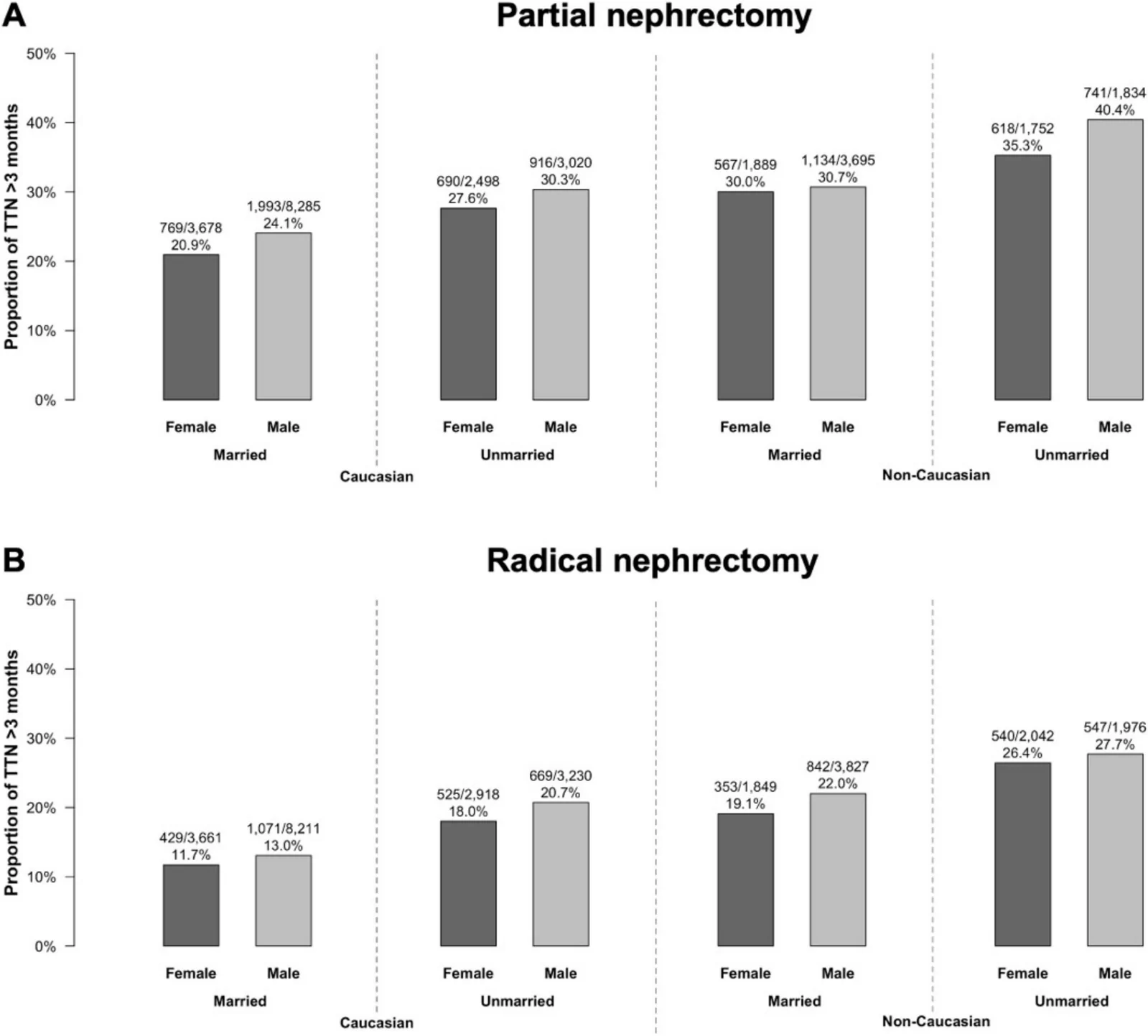
Proportion of patients with time to nephrectomy (TTN) > 3 months according to race/ethnicity, marital status and sex among patients with localized renal cell carcinoma treated with partial **A** or radical **B** nephrectomy (SEER 2004–2022)

When combining race/ethnicity, marital status, and sex, the highest proportion of TTN > 3 months was recorded in non-Caucasian unmarried males (40.4%), followed by non-Caucasian unmarried females (35.3%, Fig. 2). Intermediate proportions were observed in non-Caucasian married and Caucasian unmarried patients of both sexes (range 27.6–30.7%). Finally, a lower proportion was recorded in Caucasian married males (24.1%), whereas the lowest proportion was recorded in Caucasian married females (20.9%).

In multivariable logistic regression models, the same combinations were tested relative to the group with the lowest proportion of TTN > 3 months (Caucasian married females). In these analyses, all remaining combinations achieved independent predictor status for TTN > 3 months. Odds ratios decreased in a stepwise and proportionate fashion from the highest value of 2.70 (95% CI 2.38–3.06; p < 0.001) in non-Caucasian unmarried males to the lowest value of 1.20 (95% CI 1.10–1.33; p < 0.001) in Caucasian married males (Table 3).

### Sociodemographic determinants of TTN > 3 months in RN

In patients treated with RN, TTN > 3 months was more frequent in non-Caucasian vs. Caucasian patients (23.5% vs. 14.9%, p < 0.001), in unmarried vs. married individuals (22.4% vs. 15.4%, p < 0.001), and in males vs. females (18.1% vs. 17.6%, p = 0.3, Table 2), in that descending order. In multivariable logistic regression models, all three sociodemographic risk variables, namely non-Caucasian race/ethnicity (OR 1.74, 95% CI 1.63–1.86, p < 0.001), unmarried status (OR 1.58, 95% CI 1.48–1.69, p < 0.001), and male sex (OR 1.20, 95% CI 1.12–1.28, p < 0.001), achieved independent predictor status for TTN > 3 months (Table 3).

When combining race/ethnicity, marital status, and sex, the highest proportion of TTN > 3 months was recorded in non-Caucasian unmarried males (27.7%), followed by non-Caucasian unmarried females (26.4%, Fig. 2). Intermediate proportions were observed in non-Caucasian married and Caucasian unmarried patients of both sexes (range 18.0–22.0%). Finally, a lower proportion was recorded in Caucasian married males (13.0%), whereas the lowest proportion was recorded in Caucasian married females (11.7%).

In multivariable logistic regression models, the same combinations were tested relative to the group with the lowest proportion of TTN > 3 months (Caucasian married females). In these analyses, all remaining combinations achieved independent predictor status for TTN > 3 months. Odds ratios decreased in a stepwise and proportionate fashion from the highest value of 3.09 (95% CI 2.67–3.57; p < 0.001) in non-Caucasian unmarried males to the lowest value of 1.14 (95% CI 1.02–1.29; p = 0.029) in Caucasian married males (Table 3).

### Sensitivity analyses

Sensitivity analyses using alternative TTN thresholds (> 2 and > 4 months) and upper truncation (> 3–6 vs. ≤ 3 months) yielded consistent results across both PN and RN cohorts (Suppl. Tables 1, 2). Specifically, the direction and magnitude of the associations between sociodemographic characteristics and TTN remained stable across all analyses. Non-Caucasian unmarried patients consistently exhibited the highest risk estimates, whereas Caucasian married females represented the lowest-risk group.

## Discussion

Prolonged TTN has been confirmed as a risk factor in analyses addressing cancer control outcomes [10–18]. However, temporal trends in TTN and the role of sociodemographic characteristics as determinants of longer TTN in localized RCC, remain insufficiently characterized. We addressed these knowledge gaps within the SEER database (2004–2022) and formulated three hypotheses. We hypothesized that TTN has increased over time, that sociodemographic characteristics represent independent determinants of prolonged TTN, and that their combined assessment allows improved stratification, enabling identification of individuals at particularly high risk for prolonged TTN. Several noteworthy observations were made.

First, we relied on a large, contemporary, population-based cohort of 54,365 patients from the United States. Prior studies examining TTN were based on smaller sample sizes and focused on the association between prolonged TTN and cancer control outcomes. Specifically, the two largest previous population-based cohorts included 30,763 patients from England (2000–2010) and 22,653 patients from the United States (2004–2014) [10, 11]. Other studies relied on single-center cohorts (n = 489–1484) with limited generalizability [12–14]. Consequently, the current study provides the most comprehensive contemporary population-based estimates of TTN and is the first to explicitly focus on temporal trends and sociodemographic determinants of prolonged TTN in surgically treated patients with localized RCC.

Second, the proportion of TTN > 3 months differed according to nephrectomy type. Specifically, TTN > 3 months was observed in 28% of patients treated with PN, whereas in those treated with RN, the corresponding rate was 18%. This observation validates the need for separate analyses in PN and subsequently in RN patients. No previous large-scale study relied on this methodology. Only one single-center study by Mano et al. (1995–2013) reported separate rates of TTN > 3 months in PN (29%) and RN (16%) patients, consistent with the population-based estimates of the present study [12].

Third, the current study revealed significant temporal increase in the proportion of TTN > 3 months in PN as well as in RN patients. Specifically, rates increased from 19 to 36% at PN and from 11 to 31% at RN, over the study period (2004–2022). These observations are worrisome, since TTN > 3 months is associated with worse cancer control outcomes in localized RCC patients [10–18]. Moreover, these temporal trends suggests that TTN > 3 months, and its detrimental oncologic impact, may affect even larger proportions of patient in the future. This underscores the relevance of the subsequent parts of the present study, in which determinants of TTN > 3 months were evaluated. To the best of our knowledge, this investigation represents the first to document nephrectomy-type-specific temporal increases in TTN. Nevertheless, these findings are consistent with previous studies reporting increasing TTN over time (up to 2014) for nephrectomy in general [10, 11]. Although the underlying causes cannot be determined within the present study, several factors may have contributed to the observed trend, including evolving use of active surveillance, changes in case complexity, referral pathways and centralization of care, as well as healthcare system constraints during the COVID-19 pandemic.

Fourth, the current study identified three sociodemographic determinants of TTN > 3 months prior to both PN and RN. Specifically, in patients treated with either PN or RN, non-Caucasian race/ethnicity (PN: OR 1.49; RN: OR 1.74), unmarried status (PN: OR 1.43; RN: OR 1.58), and male sex (PN: OR 1.18; RN: OR 1.20) were independently associated with higher odds of TTN > 3 months, although the association for male sex was comparatively modest. These findings are consistent with previous investigations reporting sociodemographic disparities in TTN, although these were not the primary focus of those studies and only partially reached statistical significance. In a population-based analysis using the National Cancer Database, Zeng et al. reported higher odds of TTN > 3 months for African American (OR 1.54) and Hispanic (OR 1.44) compared to Caucasian patients, as well as for male patients (OR 1.10) [11]. Similarly, in a single-center study, Mano et al. observed higher rates of TTN > 3 months in non-Caucasian compared to Caucasian patients (OR 2.52), whereas differences according to sex were less pronounced (OR 1.11) [12]. Several explanations may account for these findings. Racial and ethnic disparities in TTN may be related to differences in referral patterns or systemic factors within the healthcare system in the United States [23]. The observed sex differences, particularly in PN, may reflect differences in healthcare-seeking behavior and access to care. Prior studies have shown that women more frequently experience barriers to healthcare access related to caregiving responsibilities, as well as differences in healthcare utilization and clinical management [24, 25]. Marital status has been less frequently examined in this context but may serve as a proxy for social support. Previous studies have demonstrated that being married is associated with improved healthcare navigation, treatment adherence, and timely access to care [26–28].

Fifth, the combined assessment of race/ethnicity, marital status, and sex allowed refined stratification of patients according to risk of TTN > 3 months by yielding eight distinct patient groups. To our knowledge, this represents the first study to report such combined sociodemographic stratification for TTN. The highest risk for TTN > 3 months was observed in non-Caucasian unmarried males (PN: 40.4%, RN: 27.7%), whereas the lowest risk was recorded in Caucasian married females (PN: 20.9%, RN: 11.7%). All combination groups achieved independent predictor status, with ORs decreasing in a stepwise and proportionate fashion from the highest values in non-Caucasian unmarried males (PN: 2.70, RN: 3.09) to the lowest values in Caucasian married males (PN: 1.20, RN: 1.14), relative to Caucasian married females. These findings indicate that sociodemographic characteristics do not act in isolation but rather exert cumulative effects, resulting in a gradient of risk across combination groups. Such patterns are consistent with the concept that multiple social determinants of health may amplify disparities in access to care [29]. Importantly, the sociodemographic disparities identified in the present study persisted despite adjustment for age, tumor size, income, and area of residence. Moreover, virtually the same results were recorded when applying alternative TTN thresholds (> 2 vs. ≤ 2 months, and > 4 vs. ≤ 4 months) as well as after upper truncation (> 3–6 vs. ≤ 3 months), indicating that the associations between specific combinations of sociodemographic risk variables and TTN are generalizable across different definitions of prolonged TTN in both nephrectomy types.

Taken together, the current findings demonstrate that TTN > 3 months has become increasingly common in localized RCC, underscoring the growing clinical relevance of this endpoint, which has convincingly been linked to adverse cancer control outcomes in previous studies [10–18]. Moreover, TTN > 3 months rates differed according to nephrectomy type, supporting separate assessment of PN an RN. TTN > 3 months was influenced by sociodemographic characteristics, namely race/ethnicity, marital status and sex. Finally, combined sociodemographic stratification enabled identification of the highest-risk patient groups. From a clinical and healthcare system perspective, these findings highlight the need to consider sociodemographic factors, both individually and in combination, when evaluating access to timely surgical care. Efforts to reduce TTN > 3 months may consider prioritizing patients at particularly high risk, namely non-Caucasian unmarried males. Thereby, the target population for intervention can be reduced, improving feasibility and potentially reducing resource utilization, including personnel effort and associated costs [30]. Ultimately, by avoiding a TTN > 3 months in these patients, the adverse effects of delaying surgery on cancer control may be mitigated [10–18].

Several limitations require acknowledgment. First, our study shares the inherent constraints of retrospective analyses based on large-scale data repositories such as SEER or National Cancer Database [11, 19, 31]. Second, SEER lacks information on the underlying reasons for prolonged TTN, and it is possible that patient preference or preoperative medical optimization accounted for some delays. Third, variables such as comorbidity burden, insurance status, laboratory values, and longitudinal assessment of tumor size are not captured in current releases of SEER, precluding efforts for more detailed analyses and potentially resulting in residual confounding. Finally, the generalizability of our findings may be limited to healthcare systems comparable to that of the United States. However, this restriction reflects the objective of the present study, which was to evaluate temporal trends and sociodemographic determinants of TTN within the United States.

## Conclusion

TTN > 3 months has become increasingly common in localized RCC, underscoring the growing clinical relevance of this endpoint. In both PN and RN, prolonged TTN was influenced by sociodemographic characteristics, namely race/ethnicity, marital status, and sex. The combined assessment of these factors enabled the identification of non-Caucasian unmarried males as patients at highest risk for prolonged TTN. These findings highlight the need to consider sociodemographic characteristics when evaluating access to timely surgical care of localized RCC. Efforts aimed at minimizing TTN could preferentially focus on non-Caucasian unmarried male individuals.

## Supplementary Information

Below is the link to the electronic supplementary material.

Supplementary file1 (PDF 371 KB)

## Data Availability

The used data are derived from the Surveillance, Epidemiology, and End Results (SEER) Program database of the National Cancer Institute. Access to SEER data is available upon request through the SEER*Stat software after registration and approval by the National Cancer Institute. The authors had authorized access to these data. The data are not publicly available due to privacy and confidentiality restrictions imposed by the SEER program.
