# Supplementary material for: Temporal trends and sociodemographic determinants of time to nephrectomy in localized renal cell cancer
Source: Int Urol Nephrol. 2026 Jul 23;58(12):5371–9. doi: 10.1007/s11255-026-05303-0 (PMC13768357; doi:10.1007/s11255-026-05303-0)
Supplement: Supplementary file 1 [file 11255_2026_5303_MOESM1_ESM.pdf]

Suppl. Figure 1. Distribution of time to nephrectomy (TTN) among patients with localized renal cell carcinoma (SEER 2004–2022)

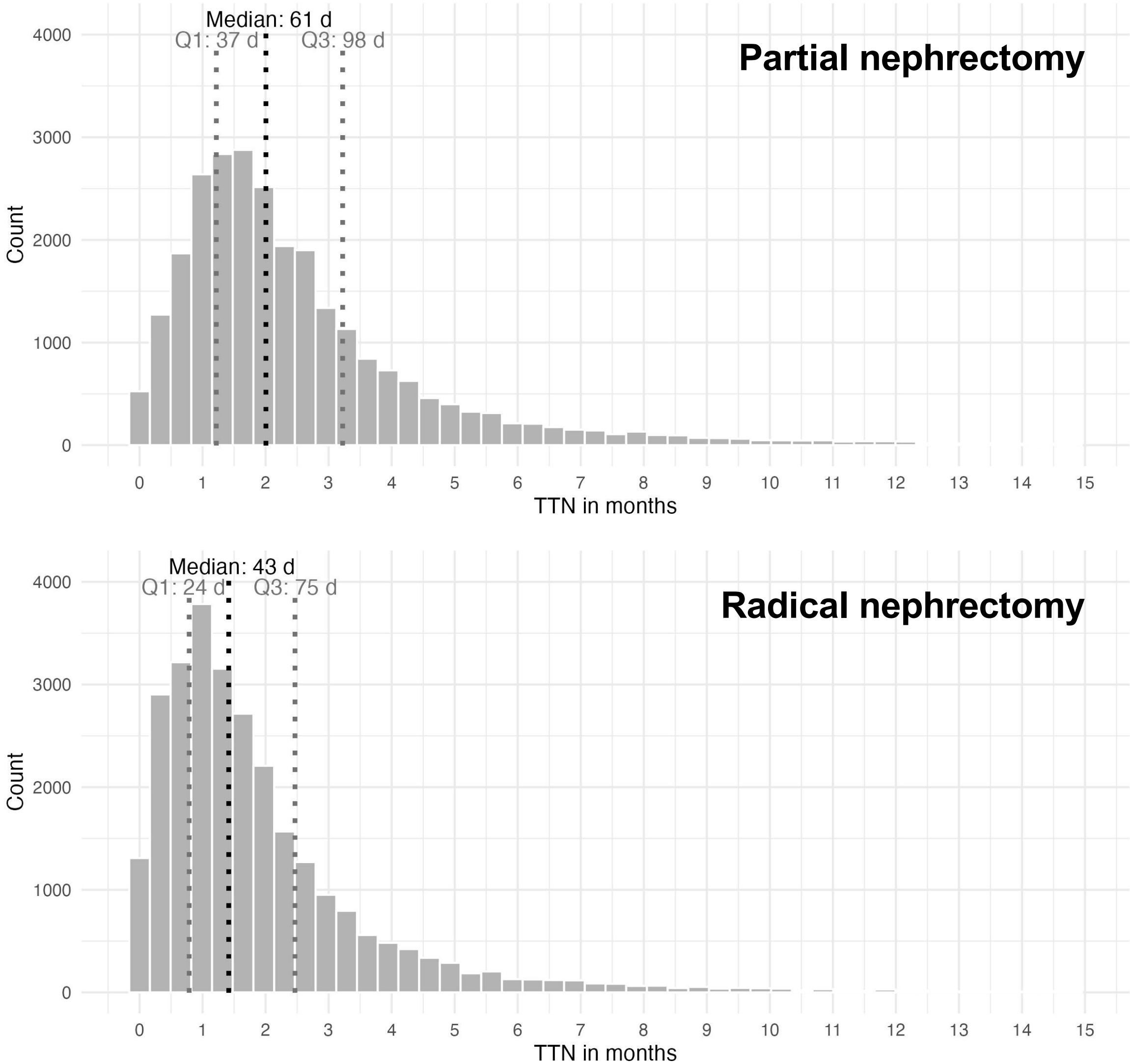

Suppl. Table 1. Multivariable logistic regression models of sociodemographic characteristics associated with time to nephrectomy >2 months (vs. ≤2 months) and >4 months (vs. ≤4 months) in patients with localized renal cell carcinoma (SEER 2004–2022)

Time to nephrectomy	>2 months (vs. ≤2 months)						>4 months (vs. ≤4 months)					
	Partial nephrectomy			Radical nephrectomy			Partial nephrectomy			Radical nephrectomy		
	OR	95% CI	p-value	OR	95% CI	p-value	OR	95% CI	p-value	OR	95% CI	p-value
**Caucasian married females**	Ref.			Ref.			Ref.			Ref.		
**Caucasian married males**	1.10	1.02–1.19	**0.016**	1.11	1.02–1.22	**0.023**	1.22	1.08–1.37	**0.001**	1.09	0.93–1.28	0.3
**Caucasian unmarried females**	1.28	1.15–1.42	**<0.001**	1.46	1.30–1.62	**<0.001**	1.51	1.30–1.74	**<0.001**	1.52	1.27–1.82	**<0.001**
**Caucasian unmarried males**	1.53	1.39–1.69	**<0.001**	1.95	1.75–2.16	**<0.001**	1.73	1.51–1.99	**<0.001**	2.19	1.86–2.59	**<0.001**
**Non-Caucasian married females**	1.54	1.38–1.73	**<0.001**	1.83	1.61–2.07	**<0.001**	1.61	1.38–1.89	**<0.001**	1.88	1.54–2.28	**<0.001**
**Non-Caucasian married males**	1.59	1.45–1.75	**<0.001**	2.09	1.89–2.32	**<0.001**	1.75	1.54–2.00	**<0.001**	2.28	1.94–2.68	**<0.001**
**Non-Caucasian unmarried females**	1.87	1.67–2.11	**<0.001**	2.34	2.07–2.63	**<0.001**	2.02	1.73–2.35	**<0.001**	2.62	2.19–3.13	**<0.001**
**Non-Caucasian unmarried males**	2.33	2.07–2.62	**<0.001**	2.89	2.56–3.25	**<0.001**	2.70	2.34–3.13	**<0.001**	3.18	2.66–3.80	**<0.001**
adjusted for year of diagnosis, age, income, area of residence, and tumor size.												

Suppl. Table 2. Multivariable logistic regression models of sociodemographic characteristics associated with time to nephrectomy >3–6 months (vs. ≤3 months) in patients with localized renal cell carcinoma (SEER 2004–2022)

**Time to nephrectomy >3–6 months (vs. ≤3 months)**	**Partial nephrectomy**			**Radical nephrectomy**		
	**OR**	**95% CI**	**p-value**	**OR**	**95% CI**	**p-value**
**Caucasian married females**	Ref.			Ref.		
**Caucasian married males**	1.21	1.09–1.34	**<0.001**	1.14	1.00–1.30	0.1
**Caucasian unmarried females**	1.40	1.22–1.60	**<0.001**	1.53	1.31–1.79	**<0.001**
**Caucasian unmarried males**	1.62	1.43–1.84	**<0.001**	1.97	1.70–2.29	**<0.001**
**Non-Caucasian married females**	1.56	1.35–1.80	**<0.001**	1.74	1.46–2.07	**<0.001**
**Non-Caucasian married males**	1.61	1.43–1.82	**<0.001**	2.01	1.74–2.32	**<0.001**
**Non-Caucasian unmarried females**	1.89	1.64–2.18	**<0.001**	2.30	1.95–2.70	**<0.001**
**Non-Caucasian unmarried males**	2.44	2.12–2.81	**<0.001**	2.73	2.32–3.22	**<0.001**

adjusted for year of diagnosis, age, income, area of residence, and tumor size.
